# Potential Mechanism of Action of Cyclosporin A in Human Dermal Fibroblasts—Transcriptomic Analysis of *CYPs*

**DOI:** 10.3390/molecules23071642

**Published:** 2018-07-05

**Authors:** Grażyna Janikowska, Alina Pyka-Pająk, Tomasz Janikowski, Jolanta Adamska, Urszula Mazurek, Przemysław Jędrusik

**Affiliations:** 1 Department of Analytical Chemistry, Medical University of Silesia, Jagiellońska 4, 41-200 Sosnowiec, Poland; apyka@sum.edu.pl; 2 Department of Molecular Biology, Medical University of Silesia, Jedności 8, 41-200 Sosnowiec, Poland; tomekjanikowski@wp.pl (T.J.); jolaa@sum.edu.pl (J.A.); umazurek@sum.edu.pl (U.M.); pjedrusik@sum.edu.pl (P.J.); 3 Department of Biomedical Computer Systems, University of Silesia, Będzińska 39, 41-205 Sosnowiec, Poland

**Keywords:** cyclosporin A, cytochrome P450 gene, fibroblasts, microarray, MTT test, SOFM

## Abstract

Effect of cyclosporin A (CsA) in a therapeutic concentration, on the expression of cytochrome P450 genes (*CYPs*), was investigated in normal human dermal fibroblast cells. The expression of 57 genes, encoding cytochrome P450 isoforms, was estimated using the microarray method. Amongst 396 normalized fluorescence signals related to cytochrome P450 activity, only 91 were strictly connected to *CYPs* and were analyzed using two methods: a self-organizing feature map of artificial neural networks and typical statistical analysis with significance level at *p* ≤ 0.05. Comparing the samples from fibroblasts cultured with CsA and those cultured without, up-regulated changes of *CYP19A1*, *1B1*, *7A1*, *7F1*, *17A1* and down-regulated *2D6* gene expression were observed. The mRNAs with increased changes were in the same neuron of the self-organizing feature map. All distinguished *CYPs* encode monooxygenases, which plays an important role in steroids biosynthesis and metabolism. Based on the obtained results, we can conclude that CsA in therapeutic concentration changes the expression profile of *CYPs* in human dermal fibroblasts, especially affecting genes linked to steroids synthesis and/or metabolism. It shows the potential mechanism of action of CsA in human dermal fibroblast cells.

## 1. Introduction

Cyclosporin A (CsA) is a classic immunosuppressive drug used, not only to prevent organ or tissue rejection, but also in many immunologically-mediated diseases, for example, atopic dermatitis, pyoderma gangrenosum, pemphigus, psoriasis, rheumatoid arthritis and dry eye [1,2,3]. There are good effects in patient treatment in the aforementioned diseases even in oral doses [4]. CsA is metabolized by cytochrome P450 (*CYP*), mainly by the isoform *CYP3A4*; it also works as an inhibitor of *3A4*, *2C19* and *2D6* in human liver microsomes [5].

Cytochrome P450 is a large, multifunctional superfamily of 57 genes encoding monooxygenases, which catalyze the conversion of many endobiotics and xenobiotics and have a strong clinical significance [6]. It also produces chemicals essential for homeostasis, as well as bile acids, cholesterol, steroids, lipids, vitamin D, retinoids, and biogenic amines. *CYPs* are involved in the metabolism of most drugs, chemicals and environmental pollutants, as well as endogenous substances [6,7]. The expression of *CYP* is tissue and organ selective [8]. Expression of different *CYPs* has been stated in cultured keratinocytes, Langerhans cells, fibroblasts and melanocytes [9].

CYP’s activity can be induced or inhibited by multiple endogenous and exogenous compounds [10]. Changes in expression of *CYP* genes in various skin models and cells were observed under influence of many different xenobiotics [11]. Human dermal cells are capable of metabolizing CsA [12]. Little is known about the effects of CsA on the cytochrome P450 family in human dermal fibroblasts. After oral administration, CsA reduces symptoms of psoriasis; however, it also plays a predominant role in the pathophysiology of nonmelanoma skin cancer in kidney transplant recipients [13]. Human skin fibroblasts are vital machinery for local production, conversion, metabolism and/or inactivation of many endogenic substances such as neurotransmitters, neuropeptides and typical hormones; cytochrome P450 is a key enzyme in these reactions [14,15]. This makes dermal fibroblasts a relevant model to investigate these genes under the influence of therapeutic CsA concentration. To enable a wide and accurate view of the cytochrome P450 family gene expression in human dermal fibroblasts, a microarray was used as a method of choice. A neural network of self-organizing feature maps was used to analyze the results, and these were confirmed by classical methods. The use of this allowed to select only the *CYPs*, which are activated by CsA in the dermal fibroblasts.

Thus, the aim of this study was to estimate the effect of CsA on the *CYP’s* expression in normal human dermal fibroblast cells.

## 2. Results

**Figure 1:**
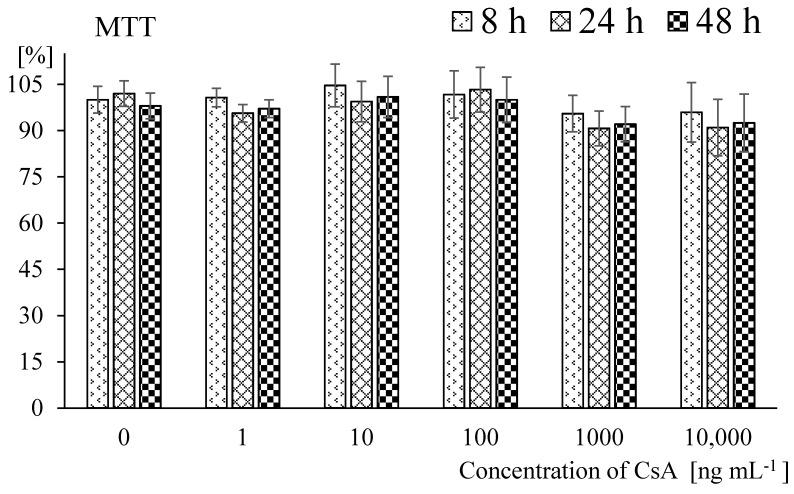
Cell viability after exposure to cyclosporin A (CsA). Legend: Exposure to different concentration of CsA for 8, 24 and 48 h in dermal fibroblasts estimated by the MTT assay (the results are showed as percent of the absorbance in control samples, mean ± SD; and lack of significance, >0.05).

The examined amounts of CsA (in the tested range of concentrations) had not shown a toxic effect on fibroblasts in MTT (Microculture Tetrazolium Test) test and results are presented in Figure 1.

Based on these results, a concentration of 100 ng per 1 mL of medium was used for future analysis, which was both comparable to the standard therapeutic dose in patients’ blood as well as nontoxic.

**Figure 2:**
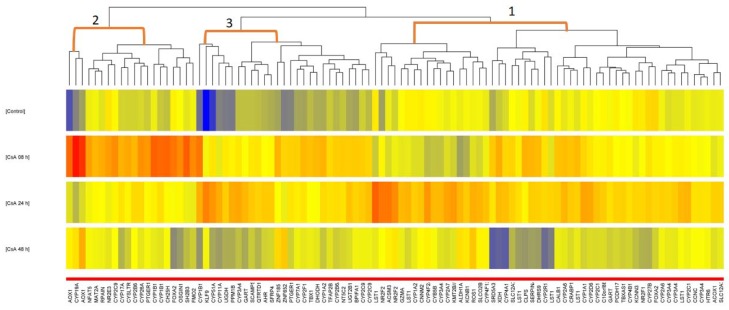
Distribution of 101 (from 396) significant transcripts linked to cytochrome P450 activity in human dermal fibroblast cells in different time of exposure to CsA. Legend: Self-organizing feature map visualized in vertical cluster tree-entities, and in horizontal conditions (three samples per each condition (0 control; 8, 24, 48 h CsA); heat maps show the color range of differences in classification of normalized fluorescence signals. The values are visualized from red (high) to blue (low).

The obtained gene expression results were used to perform two analyses. The first approach was by creating clusterization with a Kohonen map (SOFM) [16], and the second by classical statistical analysis. The next step was to filter the set of entities for statistical significance with ANOVA, and then evaluate the distribution of 101 (35 *CYPs*) from 396 mRNA (Table S1) fluorescence signals by creating a hierarchical clusterization with Euclidean distances, using the Ward method. For this purpose, Euclidean distances were calculated by taking two main clusters from which the left one was divided into two sub-groups. In turn, the right one was split into four sub-clusters (Figure 2). To verify the grouping and to choose the optimal neural network, the Davies-Bouldin value [17] (with mixed Gaussian distribution) was calculated. For this artificial neural network, the optimal k was for 3 neurons, with a critical value of 0.9134. The distribution graph of SOFM for the selected neurons is presented in Figure 2.

The inductive activities of CsA in the investigated fibroblasts showed in the following distinguished transcripts: *CYP19A1* (aromatase), *CYP1B1* (monooxygenase), *CYP2F1* (monooxygenase), *CYP7A1* (cholesterol 7α-hydroxylase) and *CYP17A1* (17α-hydroxylase) (criteria: *p* < 0.05 and FC > 1.2 for CsA 8 h vs. 0; Table 1).

**Figure 3:**
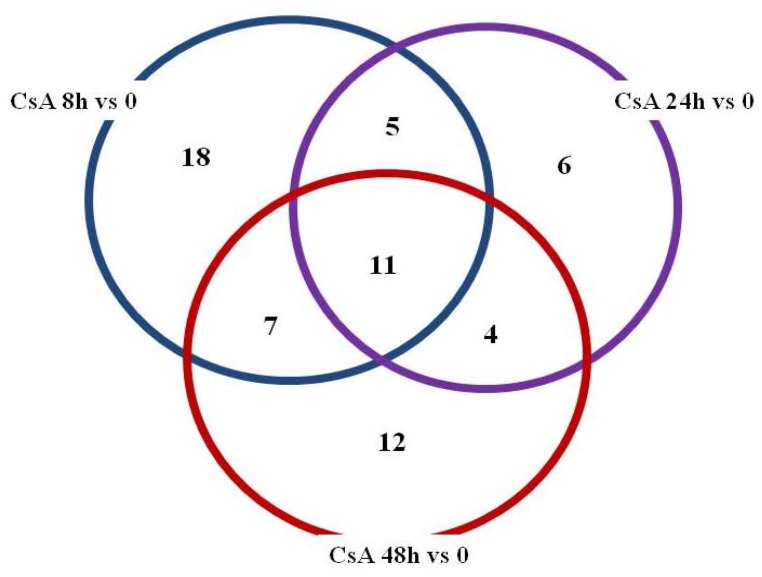
Venn diagram based on 101 transcripts selected as significant (*p* ≤ 0.05) in Tukey’s *post hoc* test from 396 linked to cytochrome P450 mRNAs at different time of exposure to CsA.

The neural networks showed a resemblance between *CYP19A1*, *2F1*, *7A1* and *17A1*, which took place in the same neuron 2, as well as *1B1* (four different probes) in both (see Table 1). The 101 mRNA from ANOVA related to *CYPs* was also analyzed using the *post hoc* Tukey’s test in all four study groups (0, 8, 24, 48 h). The analysis gave many statistically significant mRNAs for CsA: 41 for 8 h vs. 0; 26 for 24 h vs. 0; 34 for 48 h vs. 0 (Figure 3, *p* ≤ 0.05). The *CYPs* that are mentioned in Table 1 were among 18 characteristics for 8 h vs. 0 samples.

When comparing the fluorescence signals for 91 mRNAs of cytochrome P450 (Table S2) in the NHDF cultures with and without CsA, it was demonstrated that 31 mRNA of *CYPs* were up-regulated, 15 were down-regulated, and only twelve had significant changes (≤0.05, Table 2) in 8 h of exposure compared to the control.

Those from Table 1 were found with the following up-regulated mRNA encoding monooxygenases: *1A2*, *2C19* as well as *51A1* (encode sterol 14-α demethylase) from neuron 3, which are represented only by one out of two or three probes, respectively.

The analyses lead to distinguishing genes, which expression can influence the metabolism of CsA in normal human dermal fibroblasts. Additionally, Table 2 is showing 47 *CYPs* mRNA; their presence in human dermal fibroblast cells was already stated.

Taking into consideration all the methods used, the FC and value, and the expression of all probes, the six *CYPs* (*19A1*, *1B1*, *2F1*, *7A1*, *17A1* and *2D6*) were distinguished. In summary, the genes have biological significance, and directly or indirectly, are connected to biosynthesis, and/or the metabolism of steroids.

## 3. Discussion

The MTT test did not show any toxic effects of CsA in the examined concentrations on NHDFs. The result should be considered as a positive because the selection of concentration for the experiment was based on previous clinical experience. Hence, for the microarray experiment. the chosen amount was a therapeutic concentration of CsA (100 ng mL^−1^), which occurs in the circulating blood of treated patients [18,19,20]. Other findings confirm that CsA concentrations lower than 300 ng in 1 mL of medium are safe, not genotoxic nor mutagenic in normal human fibroblast cells (MRC-5) and did not affect investigated cell viability and proliferation [21]. Our findings provide a preliminary *in vitro* study that CsA does not have a significant influence on viability of fibroblast cells in 1–10,000 ng per mL range; however in therapeutic concentrations it changes the expression of *CYP* genes.

The microarray analysis provides a large-scale look inside the analyzed cells, giving a unique chance to understand the changes in response to CsA; our results are based on 396 transcripts in four cultures with and without CsA. Although gene expression does not fully show the amount of the gene product, it provides an insight into cell metabolism and indicates ongoing changes.

A number of studies which have used cell cultures, tried to explain the action and cause of side effects of CsA: among others, excessive proliferation, diabetes, hypertension, and the increased risk of non-melanoma skin cancers, especially squamous cell carcinoma [21,22,23,24]. A previous study proved that steroidogenesis and *CYPs* are involved in the pathology of skin cancer [15]. Our experiment used advanced statistical analysis, enabling a classification of main *CYPs* that may be involved in the process of dermal fibroblasts activation. The results were analyzed with the use of neural networks and a standard statistic method to calculate the impact of CsA on gene expression. The use of an artificial neural network has a wide range of usage in drug research, enabling the prediction of various processes [25]. In microarray analysis, the large amount of results can be used to create Kohonen self-organizing maps, which give the opportunity to cluster the analyzed gene expression data, showing possible interaction between them based on a mathematical algorithm [26]. The implemented clusterization illustrated a relationship between them and also highlighted the influence of CsA on the NHDF metabolism. The SOFM showed a trend in the results and indicated the resemblance in *CYP’s* expression under the influence of CsA. When looking at the distribution of the listed genes in the SOFM analysis and comparing it to their function and expression under the influence of CsA, a pattern can be seen. The created SOFM groups are a visualization of the interaction of NHDFs with CsA. It is visible that neuron 2 has higher fold change values in the first 8 h (Table 1), after which point it returns to a rather normal state. Probably these genes’ products have more specific roles and are not needed at first. As for neuron 3, the fold change of transcripts has a high level of deregulation in the first 8 h, after which they remain at an up or down regulated level (what we can see in color differences in Figure 2). It seems that the induced or inhibited genes could be strictly linked to a response to the added drug. Such data correlated with the ANOVA results not only allowed the grouping of significant genes, but also connected their expression to the effect of the drug. The distinguished genes which were different in the first 8 h of culture were assigned to adjacent neurons 2 or 3 of the Self-Organizing Feature Map (SOFM) (Figure 2; Table 1 and Table 2) and included *CYPs*: *19A1*, *1B1*, *2F1*, *7A1* and *17A1*.

*CYP19A1* gene product in the cell acts as an aromatase (also known as estrogen synthase or estrogen synthetase), a key enzyme in estrogen synthesis, which is responsible for aromatization of androgens (i.e., testosterone) into estrogens (i.e., estradiol) [27]. Similar to our findings, the presence of 19A1 was found in the fibroblast cells [28]. Additionally, authors proved that its expression was correlated with the estrogen level in the skin.

*CYP1B1* encodes ubiquitous hydroxylase, which catalyzes the hydroxylation reaction of many xenobiotics and endobiotics: these can be things such as drugs, environmental pollutants, lipids, cholesterol, and/or steroid hormones (a key enzyme in androgen metabolism to estrogen and testosterone) [6]. This gene is expressed in normal keratinocytes, melanocytes and fibroblasts as well as in induced skin cells [9,11,29]. Its expression and activity is connected to hormones that can induce some forms of cancer [27]. Our findings show the presence of *1B1* in the human dermal fibroblasts, up-regulated in response to CsA.

The *CYP2F1* gene is little known and encodes monooxygenase, which takes part in steroidogenesis.

*CYP7A1* encodes cholesterol 7α-hydroxylase, which is a crucial enzyme in the synthesis and homeostasis of cholesterol [30,31]. Products of this gene (7-hydroxylases) potentially can produce 7-hydroxy/oxy-steroids/sterols with modifying effects on local homeostasis in the skin [14]. Our results (Table 1 and Table 2) showed that *CYP7A1* is significantly increased in response to CsA.

*CYP17A1* mainly acts as gene coding pregnenolone and progesterone 17α hydroxylase and also as monooxygenase takes part in other reactions of steroidogenesis as a key enzyme, and also in drugs metabolism [6]. Its presence was found in keratinocytes and fibroblasts [30], similar to our results. Furthermore, we found that CsA stimulates its expression in human dermal fibroblasts (Table 1 and Table 2).

Genes in the third neuron of SOFM, belonging to different cytochrome P450 families such as *1A2*, *2C19* and *51A1*, were up-regulated (Table 1 and Table 2).

CYP1A2 plays a major role in the synthesis of cholesterol, steroids and lipids in the liver [6]. Low levels or amounts below the level of detection of *1A2* were found in the skin [32]. Similar to our findings, where CsA induced the *1A2*, it was shown that coal tar increased expression of this gene in the skin [33]. CYP2C19 is involved in xenobiotic metabolism (including medicines) and synthesis of cholesterol, steroids and lipids [6]. Previously, its presence was found in fibroblasts [30], similar to our findings. Additionally, our results showed an increase in expression of this gene in skin fibroblasts in response to CsA. CYP51A1 catalyzes one of the earliest stages of cholesterol biosynthesis, namely the C14-demethylation of lanosterol [34]. Our findings show that CsA in therapeutic concentration can possibly induce its expression in the fibroblast cells. As indicated in the results, these transcripts are represented only by one probe out of two or three on microarray. Thus, the mentioned results are uncertain. *CYPs* are tissue-specific genes and their expression may differ.

Primarily connected with drug metabolism, in our results, *CYP2D6* belongs to neuron 1 and is down-regulated. Similar to our findings (a decrease in response to CsA), it was shown to reduce expression of *CYP2D6* in EpiDerm™ tissues relative to full thickness human buttock skin [35]. Its expression was found in different skin cells [9,32,36,37]. The presence of all the aforementioned genes in human dermal fibroblasts has been found even in those with the weakest expression (Table 2).

In summary, the microarray analysis gives a good inside view of the cells and their reaction to the drug. Implementing an artificial neural network by clustering gene expression signals in the analyzed groups, and ANOVA with a *post hoc* test, shows that the same statistically significant genes characterize the processes in the cell in response to a stress factor like a drug being added. The fold change 1.2–1.7 for *CYP* genes may not be high but still show significant p value. Additionally, we show that the presence of highlighted CYP (*19A1*, *1B1*, *2F1*, *7A1*, *17A1*, *2D6*) in all probes on the microarray can enhance confidence to the result.

The limitation of this study is that only one type of skin cell was analyzed. It would be necessary to study keratinocytes and melanocytes in response to CsA, as it would give us a wide view of the entire skin tissue. On the other hand, this study can be advantageous, because the group is highly homogeneous. Moreover, it only affects a single view of the response to the drug and is less complicated. 

An additional limitation is the lack of validation of highlighted genes for individual time to CsA exposition by using another method. However, each gene has already been mentioned or analyzed in other studies that were discussed previously. The presence of some of these genes has been proven in fibroblasts, but not all; therefore, our findings are the first covering the entire profile of *CYP* genes in human dermal fibroblasts.

Another limitation, but perhaps an advantage, is that the analysis of the microarrays concerns only the panel of *CYPs*, without genes connected or influencing their activity. Those genes are not analyzed, because they would not be significant to the results (single significant transcript between two or three, i.e., as for *CYP2C19*). For example, in the cases shown in Figure 2, the transcription factors: *NFAT5* (nuclear factor of activated T-cells 5), *NR2E3* (nuclear receptor 2 subfamily 2 group E member 3, negatively regulating transcription) and *FOXA2* (forkhead box a2, positively regulating transcription) that belong to neuron 2, hybridized with one out of the two probes present on the microarray. The same problem applies to *KLF9* (Kruppel-like factor 9); namely two out of three transcripts had showed reverse regulation, despite *FOXA2* being a *1B1*, *2F1*, *7A1*, *17A1* transcription factor and *KLF* regulating *19A1*. Their influence cannot be estimated based on the regulation of the highlighted *CYP* genes transcription or due to their own increase or decrease, under the influence of CsA. The distribution of 396 transcripts related to cytochrome P450 led us to select only one main pathway activated by CsA in skin fibroblasts, which was steroidogenesis. The main purpose of our work was to select the *CYP* genes, which plays a major role in response to CsA in human dermal fibroblasts, and this goal has been realized. Furthermore, we can conclude that the expression profile of cytochrome P450 in human dermal fibroblasts changes in response to CsA exposition in therapeutic concentration, especially affecting genes linked to steroid synthesis and/or metabolism. One of these pathways may be directly or indirectly responsible for the development of non-melanoma skin cancer in transplant recipients who have been using cyclosporin A for many years. Could steroids be the cause? Or is the reason an increased amount of free radicals formed during the induced reactions of steroid synthesis and/or metabolism? It may be both. To answer these questions, further studies are needed.

## 4. Materials and Methods

### 4.1. Cell Culture

Normal human dermal fibroblasts-adult (NHDF-Ad, CC-2511, CloneticsTM, Lonza, Basel, Switzerland) were cultured in sterile flasks of 25 cm^2^ surface with angled neck and antibacterial filters (Nunc^®^; Sigma-Aldrich, Saint Louis, MO, USA) in initial cell density 3500 on 1 cm^2^. The cells were passaged on the sixth day and observed microscopically. To obtain a confluent monolayer, the cells were cultured in a basal fibroblast growth medium (CC-3131, FGM^TM^, Lonza) and then in a medium with standard supplements, (CC-3132, Lonza) according to the obtained producer protocol. The cell cultures were maintained at 37 °C, enriched with 5% CO_2_ of air atmosphere with constant humidity. Cells were counted microscopically in a haemocytometer.

### 4.2. Cell Viability

The cultured NHDF-Ad (CC-2511, Lonza, Basel, Switzerland) in confluent phase of growth and at a final number of 2 × 10^3^ cells per 1 well were used for the test. The cells were seeded in 96-well plates to which CsA was added at a concentration range from 1.0 to 10,000 ng per 1 mL of the medium (Sandimmun, Novartis, Basel, Switzerland) and cultured for 8, 24, 48 h in standard conditions. Each procedure included a two blank sample with a fresh medium with and without cells. After incubation time the viability assay was performed using 3-(4,5-dimethyl-2-thiazol)-2,5-diphenyl-2*H*-tetrazolium bromide (MTT) according to the standard producer’s protocol (M2128; Sigma-Aldrich). After 4 h of incubation with dye and liberation of the incorporated dye, absorbance was measured at λ = 570 nm and λ = 690 nm (reference wavelength) [38] using the MRX Revelation plate reader (Dynex Technologies, Chantilly, VA, USA). The viability test was repeated three times and there were eight replicates for each concentration of CSA.

### 4.3. Cell Culture with CsA

NHDFs (CC-2511, Lonza) were cultured in a liquid fibroblast growth medium (3132, Lonza) according to the protocol. After the confluent phase, CsA (Sandimmun, Novartis) was added to the medium at a selected concentration of 100 ng per 1 mL. The NHDFs were cultured for 8, 24 and 48 h and after each time period, the total cells were rinsed three times and harvested, then used to isolate RNA.

### 4.4. RNA Isolation

After each time period total cell RNA was isolated with the use of TRIzol reagent in accordance with the producer’s protocol (Invitrogen, Carlsbad, CA, USA). Isolated RNA was purified with RNeasy Mini Kit columns (Qiagen, Hilden, Germany). The purity of the RNA was evaluated spectrophotometrically and by using electrophoresis.

### 4.5. Microarray HGU-133A_2.0 Analysis

Eight micrograms of pure RNA were obtained and were used for preparing one microarray. Analysis was performed by cDNA and cRNA synthesis; its fragmentation and hybridization were analyzed with HG-U133A_2.0 microarrays according to the Gene Expression Analysis Technical Manual (Affymetrix, Santa Clara, CA, USA). The fluorescence intensity signal was measured using GeneArray Scanner 3000 7G (Affymetrix). During all stages, the quality of cDNA and cRNA was controlled by performing agarose electrophoresis (1%) and by absorbance measure in λ 230/260/280 nm using spectrophotometric calculator GeneQuant II (Pharmacia Biotech, Uppsala, Sweden).

All data from performed microarrays are deposited in PL-Grid Infrastructure (www.plgrid.pl/en).

### 4.6. Statistical Analysis

The cell viability results were analyzed with descriptive statistics (mean and standard deviation) and analysis of variance (ANOVA) test (with statistical significance *p* ≤ 0.05). The fluorescence signals of the 396 mRNAs obtained from cultured NHDFs with and without CsA were subjected to statistical analysis using STATISTICA 12 (StatSoft, Kraków, Poland), MATLAB 2011 (MathWorks, Natick, MA, USA) and GeneSpring 13.0 (Agilent Technologies, Inc., Santa Clara, CA, USA) software. Additionally, to achieve the goal of the study, we decided to use self-organizing maps of artificial neural networks to analyze the CYP’s among the genes linked to cytochrome P450.

The data were grouped according to the time of culturing NHDFs without (0) and with CsA (8, 24, 48 h). The clusterization process and neural networks were made for groups of 396 mRNA (Table S1) in which there were 91 *CYPs* (Table S2) and linked mRNAs that have a crucial impact on its functioning (Affymetrix NetAffx database, www.affymetrix.com). The mRNA fluorescence signals were normalized by the RMA (Robust Multichip Average; RMAExpress) method and then used in clusterization with Euclidean distance using the Ward method (STATISTICA 12) to analyze these 396 probe sets. Afterwards, the analyzed mRNA in all four groups (0, 8, 24 and 48 h) were subjected to the creation of an artificial neural network. The neural network, by choice, was the self-organizing feature map (SOFM, or Kohonen map) [16], which was trained by unsupervised learning in STATISTICA 12. An analytical estimation of SOFM grouping quality was made using the Davies-Bouldin algorithm [17], which was calculated in MATLAB 2011 version.

The standard statistical analysis was performed in GeneSpring 13.0, where descriptive statistics were calculated for all 396 mRNA with 91 *CYPs* in each group (0, 8, 24 and 48 h) and analyzed using ANOVA with the *post hoc* Tukey test. The significance level was set at *p* ≤ 0.05. From statistically significant mRNAs (*p* ≤ 0.05) with a fold change parameter (*FC* > 1.2) greater than 1.2 had been distinguished.

## 5. Conclusions

On the basis of microarray analysis of the investigated NHDF cells, CsA induces the expression of CYP19A1, 1B1, 7A1, 2F1, 17A1 and inhibits the 2D6 gene, and it can be concluded that CsA changes the expression of *CYP* genes, which can take part in steroidogenesis. Increased expression of *CYP* genes related to steroid synthesis and/or metabolism under the influence of CsA shows the mechanism of its acting in human dermal fibroblast cells.

## Figures and Tables

**Table 1:** Cytochrome P450 (*CYPs*) differentiating NHDFs (normal human dermal fibroblast) exposed on CsA in various time of culture.

Gene Symbol	Fold Change			*p* Value	SOFM Neuron	Biological Process
	8 h vs. 0	24 h vs. 0	48 h vs. 0			
*CYP19A1*	1.72	1.07	1.24	0.0006	2	steroids biosynthesis and metabolism
*CYP1B1*	1.52	1.39	1.05	0.0030	2, 3	steroids metabolism
*CYP2F1*	1.26	1.18	1.09	0.0050	2	metabolism and synthesis of lipids, cholesterol, steroids
*CYP7A1*	1.22	1.16	1.16	0.0330	2	steroids biosynthesis
*CYP17A1*	1.20	1.11	−1.04	0.0500	2	biosynthesis of cholesterol, lipids, steroids

**Table 2:** Transcripts present in human dermal fibroblasts and comparison of culture with and without CsA in the T test and the Self-Organizing Feature Map (SOFM).

No.	Probe ID	Transcript	NHDFs Control (0) Mean [log_2_FS]	NHDFs with CsA (8 h) Mean [log_2_FS]	*t* Test	SOFM Neuron
1	202436_s_at	*1B1*	12.24	12.64	↑	2
2	208131_s_at	*8A1*	12.15	12.27	NS	
3	202435_s_at	*1B1*	11.32	11.77	↑	2
4	202437_s_at	*1B1*	10.78	11.38	↑	3
5	216661_x_at	*2C19/9*	8.62	8.62	NS	
6	209975_at	*2.E1*	8.46	8.46	NS	
7	202314_at	*51A1*	8.39	8.85	↑	3
8	210726_at	*3A4*	7.96	8.03	NS	
9	219825_at	*26B1*	7.83	7.71	NS	
10	214234_s_at	*3A5*	7.81	7.90	NS	
11	216025_x_at	*2C19/9*	7.75	7.60	NS	
12	209148_at	*2C8*	7.58	7.71	NS	
13	203979_at	*27A1*	7.49	7.62	NS	
14	219565_at	*20A1*	7.47	7.49	NS	
15	205998_x_at	*3A4*	6.42	6.59	NS	
16	207244_x_at	*2A6*	6.32	6.32	NS	
17	203475_at	*19A1*	6.25	7.04	↑	2
18	220432_s_at	*39A1*	5.47	5.52	NS	
19	215103_at	*2C18*	5.47	5.34	NS	
20	214630_at	*11B2*	5.25	5.34	NS	
21	215982_s_at	*21A2*	5.23	5.33	NS	
22	214419_s_at	*2C9*	5.16	5.10	NS	
23	202434_s_at	*1B1*	5.16	5.50	↑	2
24	209976_s_at	*2.E1*	4.94	4.75	NS	
25	207498_s_at	*2D6*	4.69	4.45	↓	1
26	216607_s_at	*51A1*	4.63	4.56	NS	
27	1494_f_at	*2A6*	4.63	4.69	NS	
28	220562_at	*2W1*	4.62	4.54	NS	
29	211231_x_at	*4A11*	4.55	4.74	NS	
30	207386_at	*7B1*	4.51	4.69	NS	
31	205765_at	*3A5*	4.49	4.34	NS	
32	208327_at	*2A13*	4.49	4.61	NS	
33	211440_x_at	*3A43*	4.46	4.65	NS	
34	210576_at	*4F8*	4.35	4.29	NS	
35	211295_x_at	*2A6*	4.33	4.49	NS	
36	220331_at	*46A1*	4.32	4.44	NS	
37	210452_x_at	*4F2*	4.24	4.17	NS	
38	207609_s_at	*1A2*	4.20	4.10	NS	
39	206539_s_at	*4F12*	4.18	4.21	NS	
40	214320_x_at	*2A6*	4.17	3.88	NS	
41	204309_at	*11A1*	4.16	4.39	NS	
42	207608_x_at	*1A2*	4.14	4.44	↑	3
43	205502_at	*17A1*	4.06	4.33	↑	2
44	1431_at	*2.E1*	4.01	3.67	NS	
45	216058_s_at	*2C19*	3.17	3.40	↑	3
46	207406_at	*7A1*	2.49	2.79	↑	2
47	207913_at	*2F1*	2.51	2.86	↑	2

Abbreviations are as follows: Normal human dermal fibroblast (NHDF); hour (h); fluorescence signal (FS); no significance (NS).

## Supplementary Materials

The following are available online, Table S1: List of 396 ID mRNA and gene symbols used in the experiment; Table S2: List of 91 ID mRNA and gene symbols used in the experiment.
