# Supplementary material for: Potential Mechanism of Action of Cyclosporin A in Human Dermal Fibroblasts—Transcriptomic Analysis of CYPs
Source: Molecules. 2018 Jul 5;23(7):1642. doi: 10.3390/molecules23071642 (PMC6100361; doi:10.3390/molecules23071642)
Supplement: Supplementary file 1 [file molecules-23-01642-s001.pdf]

Potential Mechanism of Action of Cyclosporin A in Human Dermal Fibroblasts – Transcriptomic Analysis of *CYPs*

Grażyna Janikowska[1,*], Tomasz Janikowski[2], Jolanta Adamska[2], Przemysław Jędrusik[2,3], Alina Pyka-Pająk[1] and Urszula Mazurek[2]

[1] Department of Analytical Chemistry, Medical University of Silesia, Jagiellońska 4, 41-200 Sosnowiec, Poland
[2] Department of Molecular Biology, Medical University of Silesia, Jedności 8, 41-200 Sosnowiec, Poland
[3] Department of Biomedical Computer Systems, University of Silesia, Będzińska 39, 41-205 Sosnowiec, Poland
* Correspondence: gjanikowska@sum.edu.pl

Received: date; Accepted: date; Published: date

Supplementary Materials:

Table S1. List of 396 ID RNA and gene symbols of cytochrome P450 related genes.

Probe Set ID	Gene Symbol
1431_at	CYP2E1
1494_f_at	CYP2A6
1773_at	CHURC1-FNTB
200046_at	DAD1
200710_at	ACADVL
200755_s_at	CALU
200756_x_at	CALU
200757_s_at	CALU
200768_s_at	MAT2A
200769_s_at	MAT2A
200803_s_at	TMBIM6
200804_at	TMBIM6
200853_at	H2AFZ
201120_s_at	PGRMC1
201121_s_at	PGRMC1
201212_at	LGMN
201383_s_at	NBR1
201384_s_at	NBR1
201485_s_at	RCN2
201486_at	RCN2
201575_at	SNW1
201633_s_at	CYB5B
201634_s_at	CYB5B
201715_s_at	ACIN1
201790_s_at	DHCR7
201791_s_at	DHCR7
201955_at	CCNC
202139_at	AKR7A2
202152_x_at	USF2
202203_s_at	AMFR

202204_s_at	AMFR
202237_at	NNMT
202238_s_at	NNMT
202314_at	CYP51A1
	LRRD1
202329_at	CSK
202434_s_at	CYP1B1
202435_s_at	CYP1B1
202436_s_at	CYP1B1
202437_s_at	CYP1B1
202467_s_at	COPS2
202575_at	CRABP2
202814_s_at	HEXIM1
202815_s_at	HEXIM1
202820_at	AHR
202986_at	ARNT2
203180_at	ALDH1A3
203320_at	SH2B3
203343_at	UGDH
203419_at	MLL4
203472_s_at	SLCO2B1
203473_at	SLCO2B1
203475_at	CYP19A1
203541_s_at	KLF9
203542_s_at	KLF9
203543_s_at	KLF9
203585_at	ZNF185
203646_at	FDX1
203647_s_at	FDX1
203663_s_at	COX5A
203730_s_at	ZKSCAN5
203731_s_at	ZKSCAN5
203754_s_at	BRF1
203766_s_at	LMOD1
203805_s_at	FANCA
203806_s_at	FANCA
203814_s_at	NQO2
203950_s_at	CLCN6
203979_at	CYP27A1
204051_s_at	SFRP4
204052_s_at	SFRP4
204309_at	CYP11A1
204341_at	TRIM16
204616_at	UCHL3
204764_at	CHURC1-FNTB
204963_at	SSPN
204964_s_at	SSPN
205073_at	CYP2J2
205082_s_at	AOX1
205083_at	AOX1
205316_at	SLC15A2
205317_s_at	SLC15A2
205350_at	CRABP1

205355_at	ACADSB
205439_at	GSTT2
205462_s_at	HPCAL1
205480_s_at	UGP2
205488_at	GZMA
205502_at	CYP17A1
205594_at	ZNF652
205621_at	ALKBH1
205625_s_at	CALB1
205626_s_at	CALB1
205647_at	RAD52
205656_at	PCDH17
205666_at	FMO1
205676_at	CYP27B1
205749_at	CYP1A1
205765_at	CYP3A5
205776_at	FMO5
205902_at	KCNN3
205903_s_at	KCNN3
205910_s_at	CEL
205912_at	PNLIP
205939_at	CYP3A7
205942_s_at	ACSM3
205969_at	AADAC
205998_x_at	CYP3A4
205999_x_at	CYP3A4
206131_at	CLPS
206153_at	CYP4F11
206263_at	FMO4
206325_at	SERPINA6
206424_at	CYP26A1
206496_at	FMO3
206504_at	CYP24A1
206505_at	UGT2B4
206514_s_at	CYP4F2/3
206515_at	CYP4F3
206539_s_at	CYP4F12
206541_at	KLKB1
206567_s_at	PHF20
206726_at	HPGDS
206754_s_at	CYP2B6
206755_at	CYP2B6
206758_at	EDN2
206775_at	CUBN
206818_s_at	CNNM2
206929_s_at	NFIC
206932_at	CH25H
206933_s_at	H6PD
206944_at	HTR6
206985_at	HSD17B3
207000_s_at	PPP3CC
207015_s_at	ALDH1A2
207016_s_at	ALDH1A2

207122_x_at	SULT1A2
207149_at	CDH12
207155_at	TBX5
207177_at	PTGFR
207225_at	AANAT
207244_x_at	CYP2A6
207248_at	KCNA4
207386_at	CYP7B1
207392_x_at	UGT2B15
207406_at	CYP7A1
207407_x_at	CYP4A11
207416_s_at	NFATC3
207498_s_at	CYP2D6
207544_s_at	ADH6
207569_at	ROS1
207608_x_at	CYP1A2
207609_s_at	CYP1A2
207650_x_at	PTGER1
207656_s_at	ACOX1
207662_at	TBX1
207718_x_at	CYP2A7
207773_x_at	CYP3A43
207786_at	CYP2R1
207810_at	F13B
207813_s_at	FDXR
207843_x_at	CYB5A
207913_at	CYP2F1
207981_s_at	ESRRG
207998_s_at	CACNA1D
208003_s_at	NFAT5
208006_at	FOXI1
208110_x_at	MED25
208126_s_at	CYP2C18
208130_s_at	TBXAS1
208147_s_at	CYP2C8
208177_at	SLC34A1
208327_at	CYP2A13
208354_s_at	SLC12A3
208367_x_at	CYP3A4
208378_x_at	FGF5
208385_at	NR2E3
208388_at	NR2E3
208445_s_at	BAZ1B
208456_s_at	RRAS2
208606_s_at	WNT4
208647_at	FDFT1
208928_at	POR
208979_at	NCOA6
209009_at	ESD
209119_x_at	NR2F2
209120_at	NR2F2
209121_x_at	NR2F2
209155_s_at	NT5C2

209296_at	PPM1B
209352_s_at	SIN3B
209366_x_at	CYB5A
209422_at	PHF20
209423_s_at	PHF20
209471_s_at	FNTA
209505_at	NR2F1
209506_s_at	NR2F1
209600_s_at	ACOX1
209601_at	ACOX1
209620_s_at	ABCB7
209711_at	SLC35D1
209712_at	SLC35D1
209713_s_at	SLC35D1
209782_s_at	DBP
209783_at	DBP
209807_s_at	NFIX
209838_at	COPS2
209844_at	HOXB13
209874_x_at	CNNM2
209966_x_at	ESRRG
209975_at	CYP2E1
209976_s_at	CYP2E1
209990_s_at	GABBR2
209991_x_at	GABBR2
210005_at	GART
210096_at	CYP4B1
210103_s_at	FOXA2
210108_at	CACNA1D
210272_at	CYP2B7P1
210301_at	XDH
210310_s_at	FGF5
210311_at	FGF5
210328_at	GNMT
210377_at	ACSM3
210452_x_at	CYP4F2/3
210505_at	ADH7
210555_s_at	NFATC3
210556_at	NFATC3
210576_at	CYP4F8
210629_x_at	LST1
210630_s_at	RAD52
210702_s_at	PTGIS
210726_at	CYP3A4
210950_s_at	FDFT1
211006_s_at	KCNB1
211231_x_at	CYP4A11
211273_s_at	TBX1
211274_at	TBX1
211295_x_at	CYP2A6
211313_s_at	BAZ1B
211385_x_at	SULT1A2
211440_x_at	CYP3A43

211441_x_at	CYP3A43
211442_x_at	CYP3A43
211557_x_at	SLCO2B1
211562_s_at	LMOD1
211581_x_at	LST1
211582_x_at	LST1
211679_x_at	GABBR2
211726_s_at	FMO2
211843_x_at	CYP3A7-CYP3AP1
211886_s_at	TBX5
211892_s_at	PTGIS
211904_x_at	RAD52
212264_s_at	WAPAL
212267_at	WAPAL
212378_at	GART
212379_at	GART
212552_at	HPCAL1
212589_at	RRAS2
212590_at	RRAS2
212614_at	ARID5B
212651_at	RHOBTB1
212665_at	TIPARP
212699_at	SCAMP5
213061_s_at	NTAN1
213062_at	NTAN1
213225_at	PPM1B
213298_at	NFIC
213336_at	BAZ1B
213501_at	ACOX1
213631_x_at	DHODH
213632_at	DHODH
213820_s_at	STARD5
213911_s_at	H2AFZ
213950_s_at	PPP3CC
214018_at	GRIP1
214181_x_at	LST1
214234_s_at	CYP3A5
214235_at	CYP3A5
214259_s_at	AKR7A2
214261_s_at	ADH6
214312_at	FOXA2
214320_x_at	CYP2A6
214391_x_at	PTGER1
214419_s_at	CYP2C9
214420_s_at	CYP2C9
214421_x_at	CYP2C9
214451_at	TFAP2B
214574_x_at	LST1
214610_at	CYP11B1
214622_at	CYP21A2
214630_at	CYP11B2
214845_s_at	CALU
214879_x_at	USF2

215073_s_at	NR2F2
215092_s_at	NFAT5
215096_s_at	ESD
215103_at	CYP2C18
215274_at	SLC12A3
215300_s_at	FMO5
215424_s_at	SNW1
215530_at	FANCA
215633_x_at	LST1
215676_at	BRF1
215677_s_at	BRF1
215686_x_at	TFAP2B
215726_s_at	CYB5A
215737_x_at	USF2
215809_at	CYP2D6
215897_at	MED25
215982_s_at	DOM3Z
216025_x_at	CYP2C9
216058_s_at	CYP2C19
216334_s_at	CYP2A7P1
216340_s_at	CYP2A7P1
216607_s_at	CYP51A1
216661_x_at	CYP2C9
216687_x_at	UGT2B15
216720_at	CYP2U1
216961_s_at	RPAIN
216962_at	RPAIN
216990_at	GART
217021_at	CYB5A
217042_at	RDH11
217069_at	MLL4
217075_x_at	MLL4
217077_s_at	GABBR2
217133_x_at	CYP2B6
217175_at	UGT2B15
217218_at	WAPAL
217319_x_at	CYP4A22
217445_s_at	GART
217468_at	CYP2D6
217530_at	SLC34A1
217558_at	CYP2C9
217735_s_at	EIF2AK1
217736_s_at	EIF2AK1
217751_at	GSTK1
217775_s_at	RDH11
217776_at	RDH11
217813_s_at	SPIN1
217971_at	LAMTOR3
218083_at	PTGES2
218120_s_at	HMOX2
218121_at	HMOX2
218143_s_at	SCAMP2
218410_s_at	PGP

218487_at	ALAD
218489_s_at	ALAD
218769_s_at	ANKRA2
218800_at	SRD5A3
218829_s_at	CHD7
218840_s_at	NADSYN1
218964_at	ARID3B
219066_at	PPCDC
219178_at	QTRTD1
219207_at	EDC3
219240_s_at	C10orf88
219255_x_at	IL17RB
219413_at	ACBD4
219475_at	OSGIN1
219547_at	COX15
219565_at	CYP20A1
219775_s_at	CPLX3
219799_s_at	DHRS9
219825_at	CYP26B1
219835_at	PRDM8
219878_s_at	KLF13
219903_s_at	CYP2C8
220017_x_at	CYP2C9
220087_at	BCMO1
220317_at	LRAT
220331_at	CYP46A1
220420_at	LMAN1L
220432_s_at	CYP39A1
220542_s_at	BPIFA1
220562_at	CYP2W1
220619_at	CHD7
220813_at	CYSLTR2
221002_s_at	TSPAN14
221084_at	HTR3B
221304_at	UGT1A8
221530_s_at	BHLHE41
221550_at	COX15
221892_at	H6PD
222100_at	CYP2E1
222128_at	NSUN6
222183_x_at	SNW1
32540_at	PPP3CC
32541_at	PPP3CC
39705_at	SIN3B
40284_at	FOXA2
40665_at	FMO3
202820_at	AHR
202986_at	ARNT2

Table S2. List of 91 ID mRNA and gene symbols of *CYPs*.

ID	Gene
1431_at	2.E1
1494_f_at	2A6
202314_at	51A1
202434_s_at	1B1
202435_s_at	1B1
202436_s_at	1B1
202437_s_at	1B1
203475_at	19A1
203979_at	27A1
204309_at	11A1
205073_at	2J2
205502_at	17A1
205676_at	27B1
205749_at	1A1
205765_at	3A5
205939_at	3A7
205998_x_at	3A4
205999_x_at	3A4
206153_at	4F11
206424_at	26A1
206504_at	24A1
206514_s_at	4F3/2
206515_at	4F3
206539_s_at	4F12
206754_s_at	2B6
206755_at	2B6
207244_x_at	2A6
207386_at	7B1
207406_at	7A1
207407_x_at	4A11
207498_s_at	2D6
207608_x_at	1A2
207609_s_at	1A2
207718_x_at	2A7
207773_x_at	3A43
207786_at	2R1
207913_at	2F1
208126_s_at	2C18
208130_s_at	5A1
208131_s_at	8A1
208147_s_at	2C8
208327_at	2A13
208367_x_at	3A4
209148_at	2C8
209975_at	2.E1
209976_s_at	2.E1
210096_at	4B1
210272_at	2B3
210452_x_at	4F2
210576_at	4F8

210702_s_at	8A1
210726_at	3A4
211231_x_at	4A11
211295_x_at	2A6
211440_x_at	3A43
211441_x_at	3A43
211442_x_at	3A43
211843_x_at	3A7
211892_s_at	8A1
214234_s_at	3A5
214235_at	3A5
214320_x_at	2A6
214419_s_at	2C9
214420_s_at	2C9
214421_x_at	2C9
214610_at	11B1
214622_at	21A2
214630_at	11B2
215103_at	2C18
215809_at	2D6
215982_s_at	21A2
216025_x_at	2C19/9
216058_s_at	2C19
216334_s_at	2A7P1
216340_s_at	2A7P1
216607_s_at	51A1
216661_x_at	2C19/9
216719_s_at	2U1
216720_at	2U1
217133_x_at	2B6
217319_x_at	4A11
217468_at	IID7ap
217558_at	2C9
219565_at	20A1
219825_at	26B1
219903_s_at	2C8
220017_x_at	2C9
220331_at	46A1
220432_s_at	39A1
220562_at	2W1
222100_at	2.E1
